# Temporal Associations between Tri-Ponderal Mass Index and Blood Pressure in Chinese Children: A Cross-Lag Analysis

**DOI:** 10.3390/nu14091783

**Published:** 2022-04-24

**Authors:** Yixin Cui, Fan Zhang, Hao Wang, Longzhu Zhao, Ruihan Song, Miaomiao Han, Xiaoli Shen

**Affiliations:** Department of Epidemiology and Health Statistics, School of Public Health, Qingdao University, Qingdao 266071, China; 2020021072@qdu.edu.cn (Y.C.); 2020021068@qdu.edu.cn (F.Z.); 2020021085@qdu.edu.cn (H.W.); 2021021086@qdu.edu.cn (L.Z.); 2021021094@qdu.edu.cn (R.S.); 2021021078@qdu.edu.cn (M.H.)

**Keywords:** blood pressure, tri-ponderal mass index, cross-lagged panel model, children

## Abstract

Background: No longitudinal studies have explored the relationship between tri-ponderal mass index (TMI) and blood pressure (BP) in children. This study is aimed to investigate the temporal associations between TMI and BP among children in China. Methods: A longitudinal study was carried out with Chinese children from 2014 to 2019. Data of the anthropometric examination and blood pressure were collected annually. TMI was calculated by dividing weight by the cube of height. BP was measured using a standard mercury sphygmomanometer. We investigated temporal associations between TMI and BP with a cross-lagged panel model using repeated measure data from 2014 (Wave 1), 2016 (Wave 2), and 2018 (Wave 3). Results: Results of the cross-lagged panel model showed that TMI was associated with subsequent BP. Participants with higher levels of TMI presented higher levels of BP (Wave 1: β = 0.737 for systolic blood pressure (SBP) and β = 0.308 for diastolic blood pressure (DBP), Wave 2: β = 0.422 for SBP and β = 0.165 for DBP, *p* < 0.01). In addition, children with higher BP could also present higher TMI (Wave 1: β = 0.004 for SBP and β = 0.006 for DBP, Wave 2: β = 0.003 for SBP and β = 0.005 for DBP, *p* < 0.01), but the cross-lag path coefficient indicated that the influence of TMI on BP was stronger than the influence of BP on TMI. Conclusions: There was a temporal association between TMI and BP in Chinese children. Higher TMI predicted higher subsequent BP rather than the reverse relationship.

## 1. Introduction

Hypertension in childhood is a global public health issue [1]. Several studies have suggested that hypertension in childhood is a strong predictor of adult hypertension [2,3,4]. Children with hypertension also have a greater chance of developing cardiovascular disease, cognitive impairment, and retinal changes during adolescence [5,6,7]. A meta-analysis indicated that the prevalence of elevated blood pressure in Chinese children and adolescents varied from 2.2 to 26.4%, and the pooled prevalence was 9.8% [8]. Meanwhile, the prevalence of elevated blood pressure in obese children and overweight children was much higher than that in normal children [8].

Being overweight, including obesity, is defined as an excess of body fat mass [9]. Body mass index (BMI), defined as weight divided by height squared (kg/m^2^), is widely used to diagnose overweight and obesity in populations worldwide [10]. However, the applicability of BMI in children and adolescence remains controversial. Evidence has shown that weight and height squared are not proportional during adolescent growth, thus reducing the accuracy of BMI [11,12]. To overcome this limitation, Peterson et al. proposed the tri-ponderal mass index (TMI) as a substitute for BMI in body fat screening among children and adolescents, which was calculated as weight divided by height cubed [13]. For children and adolescents, TMI was more stable with age, estimated body fat mass more accurately, and presented a lower misclassification in screening adiposity [14].

Several studies have reported the association between obesity and hypertension both in adults [15,16,17] and in children [18,19,20,21,22] based on BMI. A cross-sectional study conducted in Italian adolescents found that TMI was superior to BMI in assessing hypertension [23]. To date, however, no longitudinal study has investigated whether TMI could predict subsequent changes in blood pressure in the child. In addition, the effect of blood pressure on weight status in childhood remains unclear. To address these limitations, here, we examine the temporal relationship between TMI and blood pressure over time with a traditional cross-lagged panel model (CLPM), using longitudinal data from a survey of Yantai children’s health examinations from 2014 to 2019. CLPM can be used to explore how two or more variables affect each other over time if they are measured on two or more occasions [24,25]. 

## 2. Materials and Methods

### 2.1. Study Design and Procedures

This study used data from a longitudinal cohort survey titled “Yantai children’s health examination project”, which investigated the health and development of children. The project was carried out in all primary schools in Yantai of Shandong province from 2014 to 2019. Data of the anthropometric examination and blood pressure were collected annually. First, we observed the longitudinal changes of BMI, TMI, SBP, and DBP with age in children by a fixed cohort. A total of 46,876 students at 7 years old were recruited in the 2014 academic year as baseline and were traced for 6 consecutive years to 2019. After excluding students with incomplete data (*n* = 25,519), data of 21,357 students were analyzed. Second, we performed a cross-lagged model analysis of TMI and blood pressure using data from 2014 (Wave 1), 2016 (Wave 2), and 2018 (Wave 3). A total of 328,857 students 7–12 years old were enrolled in 2014 as baseline. After excluding students with incomplete data (*n* = 196,698), data of 132,159 students were used in this set of data analysis. This study was approved by the Ethics Committee of Qingdao University Medical College (20131109). Children’s parents signed informed consent forms.

### 2.2. Study Measures

Children’s weight, height, and blood pressure (BP) were measured by well-trained health professionals using the same anthropometric measurement procedures and tools each visit. The height and weight of the students were measured without shoes and outerwear. Height was measured to the nearest 0.1 cm using a portable height board (model TZG, Jiangyin No. 2 Medical Equipment Factory, Jiangsu Province, China). Weight was measured to the nearest 0.1 kg using a portable digital scale (Shorr Productions, Olney, MD, USA). BMI was calculated as weight (kg) divided by height squared (m^2^). Weight in kilograms was divided by the cube of height in meters to calculate TMI.

BP was measured by trained observers on the right arm of students in a quiet room, in a sitting position after resting for at least 10 min. Systolic blood pressure (SBP) and diastolic blood pressure (DBP) were measured three times consecutively using a standard mercury sphygmomanometer (model XJ11D, Shanghai Medical Instruments Co., Ltd., Shanghai, China) with appropriate cuff size, making sure that cuff size fit the length and circumference of the upper arm. The first and fifth Korotkoff sound was defined as SBP and DBP. The mean values of the 3 readings were taken for analysis.

### 2.3. Statistical Analysis

Descriptive statistics for demographic information and temporal associations among the study variables were calculated using IBM SPSS Statistics for Windows (Version 21.0). Repeated measures analysis of variance was used to assess longitudinal changes in BMI, TMI, SBP, and DBP between the six consecutive years.

To examine the temporal associations between TMI and BP over time, we performed cross-lagged panel analysis using Mplus version 7.0 (Muthén & Muthén, Los Angeles, CA, USA). In this study, TMI and BP levels were measured at three waves. Missing data were processed by full information maximum likelihood (FIML) procedures in Mplus. FIML estimation used all available data to provide robust estimates for non-normal and non-independent data [26].

The cross-lagged panel model is a structural model that examines both reciprocal and longitudinal relationships among the variables [25]. We constructed four models to identify causality: (1) Model 1 was a stability model with TMI and BP (SBP and DBP) without cross-lagged structural paths, (2) Model 2 added cross-lagged paths from TMI to BP on the basis of Model 1, (3) Model 3 extended Model 2 by adding cross-lagged paths from BP to TMI, and (4) All autoregressive and cross-lagged paths from Models 1–3 were included in Model 4. We used the following fit indices to evaluate the validity of model fit: comparative fit index (CFI) ≥ 0.95, Tucker--Lewis index (TLI) ≥ 0.95, standardized root mean residual (SRMR) ≤ 0.08, and root mean square error of approximation (RMSEA) ≤ 0.06. We considered *p* < 0.05 to be statistically significant [27,28]. The chi-square difference test was used to compare the difference between nested models.

## 3. Results

### 3.1. Stability of TMI and BMI with Age

**Figure 1:**
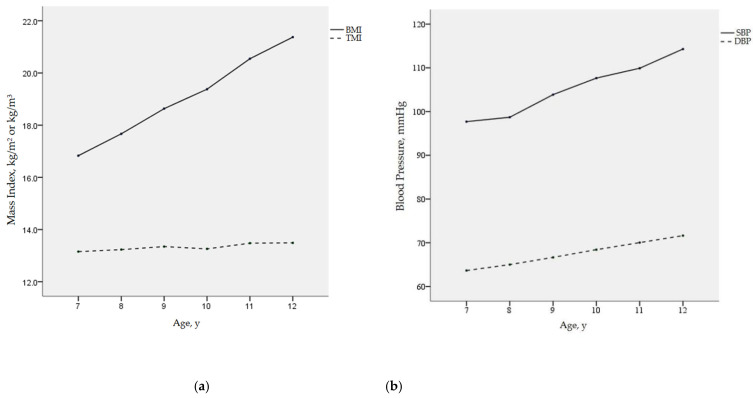
Mean values of BMI, TMI, SBP, and DBP with age: (**a**) BMI and TMI with age; (**b**) SBP and DBP with age; BMI, body mass index; TMI, tri-ponderal mass index; SBP, systolic blood pressure; DBP, diastolic blood pressure.

We investigated the longitudinal changes of BMI, TMI, SBP, and DBP with age using repeated measures analysis of variance. The results in Figure 1 show that BMI increased rapidly between ages 7 and 12 (*p* < 0.001), while TMI was almost stable throughout childhood, with population means hovering at approximately 13 kg/m^3^. In addition, both SBP and DBP levels increased with age in elementary school children (both *p* < 0.001), and SBP increased more obviously than DBP.

### 3.2. Cross-Lagged Panel Analysis

#### 3.2.1. Descriptive Statistics

We performed a cross-lagged model analysis of TMI and blood pressure using three-wave data (Wave 1, Wave 2, and Wave 3). Table 1 presents the demographic characteristics of the participants. A total of 132,159 children provided data at three waves. A total of 64,226 (48.6%) were males, and 67,933 (51.4%) were females. The mean age of participants was 9.33 years at baseline. There were significant differences between males and females in age, region, height, weight, TMI, SBP, and DBP. Compared with the previous visit, SBP and DBP were higher for each follow-up visit, suggesting an increase in blood pressure with age.

Correlations between TMI and BP across the three Waves are displayed in Table 2. Significant positive associations between TMI and SBP were found, except that SBP at Wave 1 did not correlate significantly with TMI at Waves 2 and 3. Moreover, the positive associations between TMI and DBP were explored in the three waves. The correlations of all variables were similar in three time points, indicating the stability of the relations.

#### 3.2.2. Stability Model

**Figure 2:**
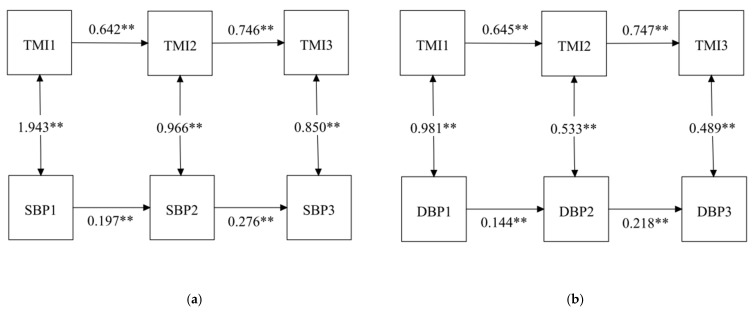
Stability model with TMI and BP among children at three time points: (**a**) stability model with TMI and SBP; (**b**) stability model with TMI and DBP. ** *p* < 0.001; TMI, tri-ponderal mass index; SBP, systolic blood pressure; DBP, diastolic blood pressure.

A stability model with TMI and BP at three waves is shown in Figure 2. After controlling sociodemographic variables, including age, sex, and region, the paths between the three waves for TMI (β = 0.642 for Wave 1 and β = 0.746 for Wave 2, *p* < 0.01) and the three waves for SBP (β = 0.197 for Wave 1 and β = 0.276 for Wave 2, *p* < 0.01) were all statistically significant. Statistical significance was also found in the three wave paths of TMI (β = 0.645 for Wave 1 and β = 0.747 for Wave 2, *p* < 0.01) and DBP (β = 0.144 for Wave 1 and β = 0.218 for Wave 2, *p* < 0.01). That is, children’s TMI and BP were stable over time. Meanwhile, TMI was significantly associated with SBP and DBP at each of the three Waves (SBP: β = 1.943 for Wave 1, β = 0.966 for Wave 2 and β = 0.850 for Wave 3, *p* < 0.01; DBP: β = 0.981 for Wave 1, β = 0.533 for Wave 2 and β = 0.489 for Wave 3, *p* < 0.01).

#### 3.2.3. Cross-Lagged Model

**Figure 3:**
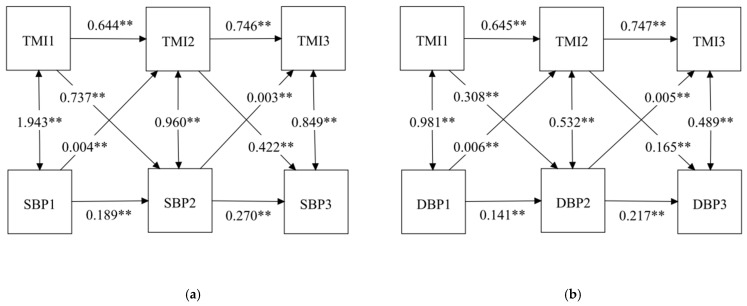
Cross-lagged model with TMI and BP among children at three time points: (**a**) cross-lagged model with TMI and SBP; (**b**) cross-lagged model with TMI and DBP; ** *p* < 0.001; TMI, tri-ponderal mass index; SBP, systolic blood pressure; DBP, diastolic blood pressure.

We analyzed the reciprocal relationship between TMI and BP by using the cross-lagged panel model. We added cross-lagged paths from TMI to BP and from BP to TMI in Models 2 and 3. Fit indices are presented in Table 3, which indicates that the models have good fits to the data. Cross-lagged paths from TMI to BP (Model 2) were significant. In addition, significant cross-lagged associations were also found between BP and TMI (Model 3). All autoregressive and cross-lagged paths were included in Model 4. The results are shown in Figure 3. A chi-square difference test between the stability model and the cross-lagged panel model was significant (χ^2^ = 2411.62 for SBP and χ^2^ = 974.82 for DBP, *p* < 0.01), suggesting that the cross-lagged model fit better. Positive and significant autoregressive coefficients indicated that all autoregressive paths had high stability over time. The cross-lagged results were similar to Models 2 and 3. In the cross-lagged panel analysis, TMI at Wave 1 was a significant predictor of BP at Wave 2 (β = 0.737 for SBP and β = 0.308 for DBP, *p* < 0.01), and TMI at Wave 2 was a significant predictor of BP at Wave 3 (β = 0.422 for SBP and β = 0.165 for DBP, *p* < 0.01). Meanwhile, BP at Wave 1 was significantly associated with TMI at Wave 2 (β = 0.004 for SBP and β = 0.006 for DBP, *p* < 0.01), and BP at Wave 2 was significantly associated with TMI at Wave 3 (β = 0.003 for SBP and β = 0.005 for DBP, *p* < 0.01). In addition, the cross-lagged path coefficients indicated that the influence of TMI on BP was stronger than that of BP on TMI.

## 4. Discussion

The longitudinal study conducted with Chinese elementary school children for the first time investigated the temporal associations between TMI and BP by cross-lagged panel model. We revealed a bidirectional causal relationship between TMI and BP. The influence of TMI on BP was stronger than that of BP on TMI. In addition, we also confirmed that TMI remained stable with age, but BMI, SBP, and DBP increased with age in elementary school children.

Several studies had reported the positive association between obesity and hypertension [29,30,31,32,33] based on BMI. Two longitudinal studies conducted separately in Guangzhou [19] and Mexico [33] both found that compared with normal weight students, overweight and obese students had a higher risk to develop hypertension. To date, however, few studies have examined the relationship between TMI and blood pressure in children [23,34]. In the present study, we demonstrated that higher TMI predicted higher blood pressure over time in Chinese children. To some extent, the results were consistent with a previous study conducted in Italy that found that TMI was associated with hypertension and was more accurate than BMI in assessing blood pressure in adolescents [23]. Since central obesity and hypertension are two criteria that define metabolic syndrome (MetS) in children and adolescents, TMI may be an indicator of detecting cardio-metabolic risk [35]. In this regard, a recent study in Colombian children, adolescents, and young adults showed a correlation between TMI and MetS [36]. Another study concluded that TMI was a better predictor of MetS than BMI in both genders [37]. In addition, our study also found that higher blood pressure in children also led to higher levels of TMI, but the causal relationship was weaker than the influence of TMI on blood pressure in children. We have noticed that no studies have previously examined the effect of BP on TMI. There is no basic experiment to explore the effect of BP on TMI, and the mechanism of BP on TMI remains unclear. The possible reason was that the strong influence of TMI on blood pressure masks the influence of blood pressure on TMI.

BMI has been used to screen for obesity. However, evidence indicates that BMI did not take into account changes in body proportions and body fat levels during adolescent growth, raising questions about the accuracy of BMI in assessing obesity in adolescents [38]. In 2017, Peterson et al. raised TMI, a super body fat index, as an alteration of BMI to assess obesity in children and adolescents for the first time. In children and adolescents, TMI was more stable over time, estimated body fat percent more accurately, and presented a lower misclassification in screening for obesity [13]. A study conducted in Finland also confirmed the high stability of TMI with age among children [39]. Consistent with the above research [13,39], our study investigated the changes of TMI and BMI in Chinese children from 7 to 12 years old and found that TMI was approximately constant at 13 kg/m^3^, while BMI increased significantly. In addition, the present study confirmed previous findings [40,41,42] that SBP and DBP increased with age in elementary school children, and the stability of SBP was higher than DBP from childhood to adulthood.

Although the underlying mechanisms between obesity and hypertension remain unclear, some possible mechanisms might explain the association. First, obesity impairs microvascular function and structure, and microvascular dysfunction may increase peripheral vascular resistance, thereby contributing to hypertension [43]. Second, several adipose tissue-derived factors may influence insulin signaling, thereby affecting insulin-mediated vasodilation, ultimately leading to hypertension [43]. Then, visceral adipose tissue produces more proinflammatory cytokines, which may induce endothelial dysfunction and play an important role in subsequent hypertension [44,45]. Finally, obesity is associated with increased adipose tissue renin and angiotensinogen, resulting in sodium retention and hypertension via activation of the renin--angiotensin system and the sympathetic nervous system [44,45].

There were several strengths in this study. First, our study was the first study to examine the bidirectional temporal relationship between TMI and blood pressure over three time points in Chinese children. Second, this study had a large sample of boys and girls from primary school, making the results representative. Finally, this study used TMI, a more accurate indicator of obesity screening in adolescents, to investigate the temporal relationship with blood pressure.

Despite the novelty strengths of this study, our study suffers from several limitations. First, this study mainly included a sample of participants from school-age children, which may limit its generalizability to a wider population. Second, several potential confounders, such as dietary factors, physical activity, parental socioeconomic status, and the educational level of the parents could influence the results of this study. However, due to data limitations, the information of the above covariates was not provided. As a result, it is impossible to adjust them. Third, we should take repeated blood pressure measurements on three different occasions, rather than three readings at one visit.

## 5. Conclusions

In summary, the longitudinal study showed that there was a dominant cross-lagged effect between tri-ponderal mass index and blood pressure, even after controlling for age, sex, and region. Higher TMI was associated with higher BP at the next time-point and subsequent time-lag, and higher BP was associated with higher TMI, but the influence of TMI on BP was stronger than that of BP on TMI. This has important implications for prevention of overweight or obesity. Our results require further prospective studies to help with the health management of children and the prevention of hypertension.

## Figures and Tables

**Table 1:** Descriptive statistics of the participants in this study.

	Total Participants	Males	Females	*p*
*N* (%)	132,159	64,226 (48.6)	67,933 (51.4)	
Age	9.33 ± 1.68	9.24 ± 1.67	9.43 ± 1.68	<0.001 ^a^
Urban, *n* (%)	66,454 (50.3)	32,256 (50.2)	34,198 (50.3)	<0.001 ^b^
Height (m)	1.41 ± 0.12	1.41 ± 0.12	1.42 ± 0.12	<0.001 ^a^
Weight (kg)	36.30 ± 9.48	36.40 ± 9.52	36.21 ± 9.44	0.001 ^a^
TMI (kg/m^3^)				
Wave 1	12.72 ± 1.69	12.90 ± 1.71	12.55 ± 1.65	<0.001 ^a^
Wave 2	12.79 ± 1.79	12.88 ± 1.88	12.70 ± 1.69	<0.001 ^a^
Wave 3	12.93 ± 1.81	12.80 ± 1.92	13.07 ± 1.69	<0.001 ^a^
SBP (mmHg)				
Wave 1	102.08 ± 12.48	102.19 ± 12.46	101.97 ± 12.50	0.001 ^a^
Wave 2	110.38 ± 12.25	111.06 ± 12.61	109.74 ± 11.88	<0.001 ^a^
Wave 3	115.04 ± 11.87	116.96 ± 12.32	113.22 ± 11.12	<0.001 ^a^
DBP (mmHg)				
Wave 1	65.32 ± 8.62	65.19 ± 8.54	65.45 ± 8.69	<0.001 ^a^
Wave 2	69.04 ± 8.72	68.80 ± 8.82	69.27 ± 8.62	<0.001 ^a^
Wave 3	72.02 ± 8.31	71.92 ± 8.53	72.12 ± 8.08	<0.001 ^a^

Values are expressed as mean ± SD, or n (%); ^a^ *p* value was calculated by T test for continuous variable; ^b^ *p* value was calculated by chi-square test for category variable.

**Table 2:** Bivariate correlations between TMI and blood pressure.

Variable	M	SD	1	2	3	4	5	6	7	8	9
1. TMI (Wave 1)	12.72	1.69	1.000								
2. TMI (Wave 2)	12.79	1.79	0.631 *	1.000							
3. TMI (Wave 3)	12.93	1.81	0.555 *	0.745 *	1.000						
4. SBP (Wave 1)	102.08	12.48	0.013 *	0.002	0.003	1.000					
5. SBP (Wave 2)	110.38	12.25	0.044 *	0.048 *	0.039 *	0.300 *	1.000				
6. SBP (Wave 3)	115.04	11.87	0.065 *	0.051 *	0.058 *	0.229 *	0.337 *	1.000			
7. DBP (Wave 1)	65.32	8.61	0.012 *	0.011 *	0.007 *	0.587 *	0.195 *	0.145 *	1.000		
8. DBP (Wave 2)	69.04	8.72	0.026 *	0.041 *	0.052 *	0.192 *	0.585 *	0.187 *	0.174 *	1.000	
9. DBP (Wave 3)	72.02	8.31	0.025 *	0.018 *	0.048 *	0.131 *	0.241 *	0.517 *	0.123 *	0.249 *	1.000

* *p* < 0.001; M, Mean; SD, Standard deviation; TMI, tri-ponderal mass index; SBP, systolic blood pressure; DBP, diastolic blood pressure.

**Table 3:** Model fit and model comparisons.

Variables		χ^2^	df	CFI	TLI	RMSEA	SRMR	Comparison	∆χ^2^	∆df
SBP	Model 1 ^a^	9050.29	20	0.966	0.923	0.058 (0.057–0.059)	0.032			
	Model 2 ^b^	6954.08	18	0.974	0.934	0.054 (0.053–0.055)	0.019	M1–M2	2096.21 **	2
	Model 3 ^c^	8723.44	18	0.967	0.917	0.060 (0.059–0.062)	0.030	M1–M3	326.85 **	2
	Model 4 ^d^	6638.67	16	0.975	0.929	0.056 (0.055–0.057)	0.018	M1–M4	2411.62 **	4
DBP	Model 1 ^a^	6349.27	20	0.971	0.936	0.049 (0.048–0.050)	0.022			
	Model 2 ^b^	5717.17	18	0.974	0.936	0.049 (0.048–0.050)	0.016	M1–M2	632.10 **	2
	Model 3 ^c^	6003.29	18	0.973	0.932	0.050 (0.049–0.051)	0.020	M1–M3	345.98 **	2
	Model 4 ^d^	5374.45	16	0.976	0.932	0.050 (0.049–0.051)	0.015	M1–M4	974.82 **	4

^a^ stability mode; ^b^ stability model with cross-lagged paths from TMI to BP; ^c^ stability model with cross-lagged paths BP to TMI; ^d^ full cross-lagged model; ** *p* < 0.01; TMI, tri-ponderal mass index; SBP, systolic blood pressure; DBP, diastolic blood pressure; BP, blood pressure; CFI, comparative fit index; TLI, Tucker--Lewis index; SRMR, standardized root mean residual; RMSEA, root mean square error of approximation.

## Data Availability

The data presented in the current study are not publicly available due to the stipulations related to privacy and confidentiality.
